# Effect of increased blood flow rate on renal anemia and hepcidin concentration in hemodialysis patients

**DOI:** 10.1186/s12882-021-02426-7

**Published:** 2021-06-15

**Authors:** Masateru Yamamoto, Tomio Matsumoto, Hiromitsu Ohmori, Masahiko Takemoto, Masanobu Ikeda, Ryo Sumimoto, Tsuyoshi Kobayashi, Akihiko Kato, Hideki Ohdan

**Affiliations:** 1 Department of Surgery, National Hospital Organization Yanai Medical Center, 95 Ihonosho, Yanai, 742-1352 Yamaguchi, Japan; 2 Department of Gastroenterological and Transplant Surgery, Applied Life Sciences, Institute of Biomedical & Health Sciences, Hiroshima University, Hiroshima, Japan; 3 Department of Pediatrics, National Hospital Organization Yanai Medical Center, Yamaguchi, Japan; 4 Blood Purification Unit, Hamamatsu University Hospital, Hamamatsu, Shizuoka Japan

**Keywords:** Anemia, Blood flow rate, Erythropoietin, Hemodialysis, Hepcidin

## Abstract

**Background:**

Increasing the blood flow rate (BFR) is a useful method for increasing Kt/V and the clearance for low molecular solutes. Hemodialysis patients are often anemic due to hypoerythropoiesis and their chronic inflammatory state. Hepcidin, a hormone that regulates iron homeostasis, is considered as an indicator of iron deficiency in patients with end-stage renal disease. This study aimed to investigate the effects of an increased BFR during hemodialysis on serum hepcidin levels and anemia.

**Methods:**

Between April 2014 and March 2016, 22 chronic dialysis patients (11 men [50.0 %]; mean [± standard deviation] age, 72 ± 12 years) undergoing maintenance hemodialysis treatment, thrice weekly, were enrolled and followed prospectively for 24 months. In April 2014, the BFR was 200 mL/min; in April 2015 this was increased to 400 mL/min, which was within acceptable limits. The dialysate flow rate remained stable at; 500mlL/min. Blood samples were collected in March 2015 and 2016. The primary endpoint was the comparison of the amounts of erythropoiesis-stimulating agent (ESA) required.

**Results:**

The increased BFR increased the Kt/V and contributed to significantly decreased urea nitrogen (UN) (*p* = 0.015) and creatinine (Cr) (*p* = 0.005) levels. The dialysis efficiency was improved by increasing the BFR. Ferritin (*p* = 0.038), hepcidin (*p* = 0.041) and high-sensitivity interleukin-6 (*p* = 0.038) levels were also significantly reduced. The ESA administered was significantly reduced (*p* = 0.004) and the Erythropoietin Resistant Index (ERI) significantly improved (*p* = 0.031). The reduction rates in UN (*p* < 0.001), Cr (*p* < 0.001), and beta-2 microglobulin (*p* = 0.017) levels were significantly greater post the BFR increase compared to those prior to the BFR increase. However, hepcidin was not affected by the BFR change.

**Conclusions:**

Increasing BFR was associated with hemodialysis efficiency, and led to reduce inflammatory cytokine interleukin-6, but did not contribute to reduce C-reactive protein. This reduced hepcidin levels, ESA dosage and ERI. Hepcidin levels were significantly correlated with ferritin levels, and it remains to be seen whether reducing hepcidin leads to improve ESA and iron availability during anemia management.

**Supplementary Information:**

The online version contains supplementary material available at 10.1186/s12882-021-02426-7.

## Background

Appropriate dialysis management is essential in reducing morbidity and mortality among patients on maintenance hemodialysis (HD) [1]. For patients with end-stage renal disease (ESRD), long-term HD can place a significant burden on quality and duration of life [2]. Long-term HD may result in treatment-related adverse effects such as blood pressure fluctuations, muscle cramps, headaches, and vomiting. Additionally, cardiovascular disease is the leading cause of death and is known to have worse outcomes in HD patients than the general population [3]. Therefore, optimizing the amount of dialysis delivered is an important consideration in high risk populations receiving burdensome treatments.

Blood flow rate (BFR) dose varies by region and facility. For example, HD patients in the USA achieve a mean BFR greater than 400 mL/min compared with BFRs that are typically less than 200 mL/min in Japan [4]. A previous study reported increased urea clearance with increased BFR when the dialysis machine, dialysis membrane, and dialysate flow rate remained constant [5]. Therefore, a lower BFR may result in insufficient dialysis and exacerbate clinical outcomes for patients with HD. However, there have been few studies on the relationship between BFR and morbidity and mortality in HD patients; thus, the optimal BFR remains unclear.

Hepcidin-25 (hepcidin) is a hepatocyte-derived small peptide hormone that regulates iron homeostasis. The hepcidin level is elevated by iron storage and inflammation, and conversely diminished in response to iron deficiency, hypoxia, and enhanced erythropoiesis [6]. Anemia is one of the most common complications of chronic kidney disease and ESRD, and is associated with lower quality of life and higher mortality. Insufficient production of erythropoietin and iron deficiency is the main cause of anemia in maintenance HD patients. The administration of erythropoiesis-stimulating agent (ESA) has significantly improved the outcomes for renal anemia. Hepcidin has been suggested as a tool for managing iron therapy in HD patients on ESAs. However, whether serum hepcidin may help clinicians in predicting and monitoring iron therapy in maintenance HD patients remains debatable.

The aim of the current study was to investigate the effect of an increased BFR on anemia in long-term HD patients.

## Materials and methods

### Study design and patients

This study was conducted in the National Hospital Organization Yanai Medical Center from April 2014 and March 2016. Overall, 22 maintenance HD patients were recruited and followed prospectively for 24 months. Patients were included if they had: undergone HD thrice weekly for at least 6 months and vascular access through arteriovenous fistula or prosthesis. Patients were excluded if they had any of the following: liver disease, malignant tumors, active collagen disease, chronic hemorrhage, and infection.

### Patient management

All patients received HD using APS-15SA, 18EA, or 21SA, high-flux dialyzers (ASAHIKASEI Medical). The endotoxin levels of dialysate were less than 0.001 EU/mL and no bacteria were found either before or after increasing BFR in purifying dialysate. The regular patient management, including iron and ESA doses was performed according to the 2008 Japanese Society for Dialysis Therapy Guideline [7]. ESA and iron supplementation were administered via the venous line at the end of a dialysis session. All patients had been receiving intravenous Epoetin Alfa (Kissei Pharmaceutical Co., Ltd. Matsumoto City, Japan). The ESA dose was adjusted to achieve the target hemoglobin within the range of 10.0–12.0 g/dL according to the recommendations of the guidelines[7]. When hemoglobin decreased to < 10 g/dL, serum TSAT to < 20 % and ferritin levels to < 100 ng, iron was supplemented by a 40 mg dose of saccharated ferric oxide (Fesin; Nichiiko Pharmaceutical, Toyama City, Japan). Blood was collected and evaluated at the beginning of the week before dialysis, and if necessary, iron and ESA were administered after dialysis. All enrolled patients were administered at least one intravenous injection of iron during the study period. Patients were anticoagulated during dialysis with 2,500-3,500 IU intravenous unfractionated heparins. The maximum blood flow in HD patients was changed to 400 mL/min within the acceptable range across the facility; no other changes were made to the HD prescriptions.

### Data collection

Baseline demographic findings including age, sex, height, weight, causes of ESRD, and therapeutic characteristics were retrieved from the hospital database and reviewed. Blood samples were collected at the start and end of each dialysis session, spaced 2 days apart from the previous dialysis session, before increasing the participants BFR. They were again collected 1 year after the BFR was increased. Red blood cell (RBC), hemoglobin (Hb), hematocrit (Hct), albumin, total cholesterol, serum urea nitrogen (UN), and creatinine (Cr) levels were determined from the blood samples. Total, free, and acylcarnitine were measured using an enzyme cycling assay. Beta-2microglobulin (b2MG) was measured using the latex agglutination method. Iron, ferritin, iron binding capacity, and transferrin were measured using the Nitro-so-PSAP. Transferrin saturation (TAST) was calculated as the serum iron/total iron binding capacity. Immunoglobulin and the cluster of differentiation (CD)4/CD8 were also tested (Bio-Medical Laboratories, Inc. Tokyo, Japan). Measurements of natural killer (NK) cell activity, soluble interleukin-2 receptor (sIL-2R), and trace elements were obtained as previously reported [8]. Serum hepcidin-25 was determined using liquid chromatography-tandem mass spectrometry (Medical Care Proteomics Biotechnology, Co., Ltd. Kahoku-gun, Japan). Single-pool Kt/V was performed monthly according to the methods reported by Daugirdas et al. [9]. The hepcidin, UN, Cr, and b2MG reduction ratios were calculated as (the value at the start of dialysis − the value at the end of dialysis) / the value at the start of dialysis. The erythropoietin resistance index (ERI) was defined as the weekly ESA dose weight-adjusted divided by the Hb level [10]. ERIs were calculated in March both in 2015 and 2016.

### Statistical analysis

Continuous variables were expressed as means and standard deviations (SDs) and were compared using Mann-Whitney U tests. Categorical variables were expressed as numbers and proportions and were compared using Fisher’s exact tests. Statistical analyses were performed using JMP Pro (version 14; SAS Institute, Cary, NC, USA). P-values < 0.05 were considered statistically significant.

## Results

The 22 patients (11 men, 50.0 %) were included. The mean age (± SD) was 72 ± 12 years and the mean (± SD) time on dialysis was 16.5 ± 12.2 years. Eleven (50.0 %), seven (31.8 %), and four (18.2 %) patients had diabetes mellitus, hypertension, and chronic glomerulonephritis, respectively.

As shown in Table 1, the mean (± SD) BFR following the BFR increase was significantly higher than that prior to the BFR increase (362 ± 34 vs. 200 mL/min; p < 0.001). After increasing the BFR without changing the treatment time, Kt/V was significantly higher in post the BFR increase, but there was no significant difference in dry weight. There were no significant differences in RBC, Hb, Hct, albumin, and total cholesterol levels pre and post the BFR increase. The UN and Cr levels were not significantly different in pre and post the BFR increase (conventional vs. increased was 45.1 ± 11.3 vs. 47.2 ± 14.8; *p* = 0.511 and 7.1 ± 2.9 vs. 6.4 ± 2.7; *p* = 0.432, respectively). Selenium was significantly increased, but no significant differences were found for other trace elements. There were no differences in iron, total iron binding capacity, unsaturated iron binding capacity, and TSAT levels. The ferritin level was significantly lower; additionally, the ESA dose, ERI, and Hepcidin were significantly reduced after increasing the BFR. Monthly changes in RBC, Hb, and Hct levels and ESA doses are shown in Supplementary Figure 1.

**Table 1:** Comparison the two groups between conventional BFR and increased BFR

	Conventional BFR	Increased BFR	*p*-value
BFR, mL/min	200	362 ± 34	< 0.001
Kt/V	1.5 ± 0.3	2.0 ± 0.4	< 0.001
Treatment time, hrs	3.9 ± 0.3	4.1 ± 0.3	0.798
BMI, kg/m^2^	18.7 ± 3.5	19.4 ± 3.4	0.385
Dry weight, kg	47.4 ± 13.1	49.3 ± 13.1	0.605
RBC x10^3^/mL	331 ± 33	325 ± 41	0.631
Hb, g/dL	10.4 ± 1.0	10.2 ± 1.0	0.589
Hct, %	31.1 ± 2.5	31.1 ± 3.2	0.897
Alb, g/dL	3.3 ± 0.5	3.2 ± 0.4	0.383
T. Chol, mg/dL	153 ± 31	162 ± 32	0.347
UN, mg/dL	45.1 ± 11.3	47.2 ± 14.8	0.511
Creatinine, mg/dL	7.1 ± 2.9	6.4 ± 2.7	0.432
b2MG	28.8 ± 4.5	25.6 ± 4.9	0.076
Copper, mg/dL	95 ± 20	85 ± 29	0.207
Zinc, mg/dL	49 ± 7	53 ± 12	0.532
Selenium, mg/L	60 ± 9	73 ± 14	0.005
Fe, mg/dL	52 ± 22	50 ± 22	0.488
Ferritin, ng/mL	188 ± 152	127 ± 114	0.038
TIBC, mg/dL	205 ± 55	215 ± 64	0.725
UIBC, mg/dL	149 ± 53	165 ± 68	0.411
TSAT, %	27.8 ± 11.8	24.5 ± 11.4	0.438
Total Carnitine, mmol/L	59.1 ± 54.3	31.8 ± 8.9	0.291
Free Carnitine, mmol/L	37.6 ± 34.4	19.7 ± 5.9	0.231
Acylcarnitine, mmol/L	21.4 ± 20.8	12.1 ± 3.7	0.526
nPCR	0.68 ± 0.11	0.76 ± 0.17	0.086
%CGR	69.1 ± 25.5	69.6 ± 31.1	0.922
Hepcidin, ng/mL	77.8 ± 83.1	46.7 ± 68.7	0.041
ESA, dose/Week	6875 ± 2720	4187 ± 2289	0.004
ERI, dose/kg/g/dL/W	14.9 ± 7.9	9.3 ± 5.7	0.031
CRP, mg/dL	0.55 ± 0.59	0.46 ± 0.71	0.185

*Alb* albumin, *BFR* blood flow rate, *b2MG* b2microglobulin, *BMI* body mass index, *CGR* creatinine generation rate, *CRP* C-reactive protein, *Hb* hemoglobin, *Hct* hematocrit, *nPCR* normalized protein catabolic rate, *RBC* red blood cell, *T. Chol* total cholesterol, *TAST* Transferrin saturation *UN* urea nitrogen

Regarding the immunological and inflammatory makers, IL-6 was significantly lower after increasing BFR (p = 0.038). In contrast, NK cell activity, sIL-2R, CD4/8 ratio, and immunoglobulin did not change pre and post the BFR increase (Table 2).

**Table 2:** Comparison the two groups between conventional BFR and increased BFR on immunological and inflammatory parameters

	Conventional BFR	Increased BFR	*p*-value
NK cell activity			
E/T ratio10:1, %	10.2 ± 8.1	13.0 ± 8.0	0.171
E/T ratio20:1, %	16.9 ± 12.5	22.1 ± 12.1	0.106
IL-6, pg/mL	13.8 ± 2.5	4.9 ± 2.6	0.038
sIL-2R, U/mL	1376 ± 1122	1349 ± 552	0.269
CD4/8 ratio	2.3 ± 3.6	1.6 ± 0.9	0.744
IgG, mg/dL	1442 (512–2500)	1484 (842–2680)	0.702
IgA, mg/dL	210 (60–479)	265 (95–580)	0.275
IgM, mg/dL	58 (22–149)	69 (27–147)	0.245

*CD* cluster of differentiation, *E/T ratio* effector cells/target cells ratio, *Ig* immunoglobulin, *IL-6* interleukin-6, *NK cell* natural killer cell, *sIL-2R* soluble interleukin-2 receptor

**Fig. 1:**
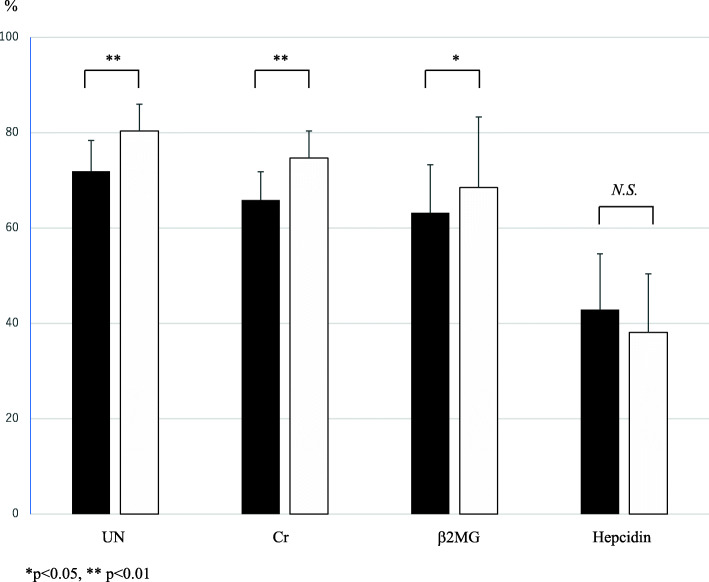
Comparison of reduction rate in UN, Cr, b2MG, and Hepcidin between the conventional and increased BFR groups The black bar represents conventional BFR, while the white bar represents increased BFR

As shown in Table 1, there was no difference between pre and post the BFR increase in UN, Cr, and b2MG before dialysis, but all decreased significantly after dialysis with increased BFR. Thus, as shown in Fig. 1, the reduction rates of post the BFR increase in the UN (80.4 ± 5.6 vs. 71.9 ± 6.5, *p* < 0.001), Cr (74.7 ± 5.7 vs. 65.9 ± 5.9, *p* < 0.001), and b2MG (68.5 ± 14.8 vs. 63.2 ± 10.1, *p* = 0.017) levels were significantly greater than those of pre the BFR increase. Small molecules that were assessed to determine dialysis efficiency, such as Kt/V, UN, and Cr, improved significantly after the BFR increase. However, there was no difference pre and post the BFR increase for hepcidin (38.1 ± 12.3 vs. 42.9 ± 11.7, *p* = 0.211).

The correlation between hepcidin and laboratory variables is shown in Table 3. No correlation was found between hepcidin and RBC, Hb, and Hct. Hepcidin was significantly positively correlated with ferritin. Hepcidin did not correlate with ESA and ERI, nor with CRP and IL-6.

**Table 3:** Pearson’s correlation of hepcidin level with laboratory parameters

	Peason’s coeffcient	*p*-value
RBC x10^3^/mL	0.224	0.332
Hb, g/dL	0.138	0.799
Hct, %	0.141	0.443
Fe, mg/dL	0.231	0.255
Ferritin, ng/mL	0.569	< 0.001
TSAT, %	0.236	0.241
ESA, dose/Week	0.125	0.497
ERI, dose/kg/g/dL/W	0.211	0.644
CRP, mg/dL	0.151	0.137
IL-6, pg/mL	0.156	0.448

*CRP* C-reactive protein, *ERI* Erythropoietin Resistant Index, *ESA* erythropoiesis-stimulating agent, *Hb* hemoglobin, *Hct* hematocrit, *IL-6* interleukin-6, *RBC* red blood cell, *TAST* Transferrin saturation

## Discussion

We found that increased BFR significantly reduced the UN, Cr, ferritin, IL-6 and hepcidin levels in HD patients included in this study. It also significantly reduced the ERI and the ESA required. The reduction rate in UN, Cr, and b2MG were significantly increased following the BFR increase; however, there was no significant difference for hepcidin.

Kt/V is well known as a commonly used marker for dialysis adequacy [1]. The 2015 Kidney Disease Outcomes Quality Initiative Guidelines recommended that per one dialysis session, Kt/V should be targeted at a minimum of 1.2, however, this is usually at least 1.4. Additionally, these sessions should be conducted thrice weekly. Kt/V can be adjusted according to dialyzer efficiency, treatment time, dialysis frequency, dialysate flow rate, and blood flow rate [11]. Based on the analyses from the Dialysis Outcomes and Practice Patterns Study (DOPPS), Kt/V is relatively low in Japan compared to that in other countries [12]. This is due to the fact that the treatment time in Japan is nearly the same as that in other countries; however, the blood flow is quite low.

The BFR is one of the most important factors in determining an appropriate Kt/V [13]. The DOPPS showed that the BFR varies depending on country [14]; thus, the optimal BFR is unclear. The impact of BFR on survival has been controversial. Urea removal is characterized by fitting mathematical kinetic models; its maintenance level has been reported to be an important factor associated with complications and deaths in dialysis patients. In order to increase the efficiency of urea removal, it is believed that increasing the BFR is effective [15]. In our study, the UN and Cr clearances were better at higher flow rates. Despite the Japan DOPPS suggesting that a higher BFR may reduce the risk of death, Japanese dialysis clinical practitioners are concerned about the increased cardiovascular burden caused by an increase in BFR and thus ensured that the BFR levels were lower. However, with BFR at 400–500 mL/min, there was neither increase in blood flow access nor were there acute changes in cardiac function or blood pressure [16, 17]. In fact, high-efficiency dialysis with a BFR greater than 450 mL/min did not increase the risk of death [18] and there was no increase in cardiac-related death in the high-dialysis group in the HEMO study in which a higher BFR was used [19]. It is considered that low BFR helps maintain high rates of AVF patency and is appropriate for the smaller Japanese body size, which may explain the longer survival observed when compared to other countries [12]. On the contrary, another study showed that increased BFR was important for the optimal dialysis dose; an insufficient dialysis dose is associated with increased mortality [20].

Hepcidin inhibits absorption via its ability to bind to ferroportin-1, which exports the cellular iron in enterocytes. In macrophages, a similar process inhibits the release of iron recycled from erythrophagocytosis [21]. Elevated hepcidin alleviates iron overload by decreasing iron absorption and increasing sequestration within the reticuloendothelial system under the condition of iron repletion [21]. Hepcidin synthesis is rapidly increased by both increased iron stores and inflammation, whereas it is decreased in iron deficiency and ineffective erythropoiesis [6]. IL-6 was higher in the conventional BFR group, thus indicating a more advanced inflammatory state than in the increased BFR group. The inflammatory cytokines, such as IL-6 induce hepcidin secretion [22]. In addition, hepcidin is cleared by filtration in the kidneys; decreased kidney function is the likely factor contributing to the rise in serum hepcidin levels. Indeed, an inverse correlation between glomerular filtration rate and serum hepcidin has been reported [23]. When the BFR was increased, the urea clearance increased, thus increasing the reduction rate of UN and Cr. In contrast, there was no difference in the reduction rate of hepcidin pre and post HD due to differences in the blood flow. A previous pilot study also found no significant difference in hepcidin clearance following dialysis [24]. The improvement of uremia that occured with increased BFR may have reduced pre-HD hepcidin by decreasing inflammation or improving erythropoiesis.

Similar to previous studies[25–27], hepcidin was significantly correlated with ferritin levels. Ferritin is a cellular iron storage molecule, the concentration of which reflects a combination of iron storage status and inflammation[28]. Ferritin levels were significantly higher post the BFR increase compared to pre, but the decrease in IL-6 did not correlate with hepcidin and CRP was not significantly decreased. Furthermore, the failure to measure erythroferrone, a potent suppressor of hepcidin expression, in this study cannot explain the potential inhibition of hepcidin. Further research is required to determine whether higher ferritin levels cause an increase in hepcidin or whether an increase in hepcidin contributes to an increase in ferritin.

The main cause of renal anemia is attributed to the lack of erythropoietin due to kidney injury. Another major determinant is the chronic inflammatory state, where cytokines further reduce erythropoietin production, induce apoptosis of erythroid precursors, and also decrease the absorption and availability of iron for erythropoiesis [29]. The result is a “functional” iron deficiency, and even with seemingly increased body iron stores, 10–20 % of cases will present with erythropoietin resistance, such as a lack of response to erythropoietin. In the present study, there was no difference in RBC, Hb or Hct levels pre and post the BFR increase. Regarding iron availability, there was no difference in Fe, TIBC, and TSAT pre and post the BFR increase; however, ferritin levels were lower after the BFR increase. The ERI was also low and there was improvement in erythropoietin resistance. Adequate dialysis achieved by excluding small, medium, and large molecules that inhibit erythropoiesis is of paramount importance to correct for anemia; increasing the reduction rate in UN has also been shown to increase Hct [30]. As shown in Fig. 1, we found that increasing the BFR increased the reduction rate in UN and made Hct comparable to that requiring higher erythropoietin doses.

There was a significant increase in selenium levels post the increase in the BFR. The cause of selenium deficiency in HD patients is a low protein dietary intake. In general, protein intake is restricted to prevent renal dysfunction as a result of excessive protein [31]. In HD patients, the question is whether selenium is lost from the dialyzer. In a previous study, the selenium concentrations measured at the inlet and outlet of the dialyzer were exactly the same [32], and therefore the involvement of adsorption on the membrane of the dialyzer and filtration from the dialyzer in selenium loss is negative. In addition, the relationship between the inflammatory status and decreased selenium level has been pointed out [33], and it is speculated that the improvement in the inflammatory status due to the increased BFR contributed to the elevated selenium level. In the aforementioned study by Koenig et al. [32], compared to individuals with normal renal functionality, HD patients had lower serum selenium levels and the selenium-containing enzyme glutathione peroxidase (GPx) was also significantly lower. Additionally, plasma levels of malondialdehyde, an index of lipid peroxidation, were higher in HD patients. GPx is thought to exert antioxidant effects by reducing hydrogen peroxide and phospholipid peroxides, acting as a protective factor against a wide range of oxidative stresses [34]. Although there was no difference in nPCR values pre and post the BFR increase, the decrease in ESA dosage may have been due to the antioxidant effect of elevated selenium levels. We speculate that this in turn stabilized the RBC membranes and prolonged the RBC life.

The present study has some limitations which merit mentioning here. First, the sample size was small, the study was conducted at single center, and the cohort consisted of Japanese patients only. Thus, it is uncertain whether our results can be generalized to other ethnic groups, especially considering the fact that the rate of AVF is high among Japanese patients. Second, although our study was a prospective observational study, patients were not randomly allocated. Future multicenter studies with larger sample sizes are warranted to infer the effects of high BFR and the variability in hepcidin levels in dialysis patients. Third, the suppression of hepcidin in this study suggests that erythroferrone was potentially elevated, but erythroferrone could not be measured at this time. Further experiments, including measurements of circulating levels of erythroferrone in HD patients, will be needed to determine its exact contribution to the pathophysiology of general anemia.

## Conclusions

An increased BFR contributed to a significant reduction in inflammatory cytokine IL-6, but not in CRP. In addition, it reduced hepcidin levels, ESA dosage and ERI. Hepcidin levels were significantly correlated with ferritin levels, and it remains to be seen whether reducing hepcidin leads to improve ESA and iron availability during anemia management.

## Supplementary Information

**Additional file 1: Supplementary Figure S1.** Monthly changes in RBC (A), Hb (B), and Hct (C) levels and ESA doses (D)

## Data Availability

The datasets generated and analyzed for the current study are not publicly available due to institute regulations, but they are available from the corresponding author on reasonable request.

## Abbreviations

b2MG: Beta-2microglobulin
BFR: Blood flow rate
CD: cluster of differentiation
Cr: Creatinine
DOPPS: Dialysis Outcomes and Practice Patterns Study
ERI: Erythropoietin Resistant Index
ESRD: End-stage renal disease
ESA: Erythropoiesis-stimulating agent
GPx: Glutathione peroxidase
Hct: Hematocrit
Hb: Hemoglobin
NK: Natural killer
RBC: Red blood cell
sIL-2R: Soluble interleukin-2 receptor
SDs: Standard deviations
TAST: Transferrin saturation
UN: Urea nitrogen

## References

[1] Held PJ, Port FK, Wolfe RA, Stannard DC, Carroll CE, Daugirdas JT (1996). The dose of hemodialysis and patient mortality. Kidney Int.

[2] Finkelstein FO, Wuerth D, Finkelstein SH (2009). Health related quality of life and the CKD patient: challenges for the nephrology community. Kidney Int.

[3] MacNeill SJ, Ford D, Evans K, Medcalf JF (2018). Chapter 2 UK Renal Replacement Therapy Adult Prevalence in 2016: National and Centre-specific Analyses. Nephron.

[4] Tentori F, Zhang J, Li Y, Karaboyas A, Kerr P, Saran R (2012). Longer dialysis session length is associated with better intermediate outcomes and survival among patients on in-center three times per week hemodialysis: results from the Dialysis Outcomes and Practice Patterns Study (DOPPS). Nephrol Dial Transplant.

[5] Hamilton G, Locking-Cusolito H (2003). Hemodialysis adequacy and quality of life: how do they relate?. Cannt j.

[6] Muckenthaler MU (2008). Fine tuning of hepcidin expression by positive and negative regulators. Cell Metab.

[7] Tsubakihara Y, Nishi S, Akiba T, Hirakata H, Iseki K, Kubota M (2010). 2008 Japanese Society for Dialysis Therapy: guidelines for renal anemia in chronic kidney disease. Ther Apher Dial.

[8] Tanaka A, Kato A, Suzuki Y, Suzuki M, Ohmori H, Sumimoto R (2014). Association of increased indoleamine 2, 3-dioxygenase with impaired natural killer cell activity in hemodialysis patients. Ther Apher Dial.

[9] Daugirdas JT (1993). Linear estimates of variable-volume, single-pool Kt/V: an analysis of error. Am J Kidney Dis.

[10] López-Gómez JM, Portolés JM, Aljama P. Factors that condition the response to erythropoietin in patients on hemodialysis and their relation to mortality. Kidney Int Suppl. 2008(111):S75-81. 10.1038/ki.2008.523 19034333

[11] Singh S, Choi P, Power A, Ashby D, Cairns T, Griffith M (2013). Ten-year patient survival on maintenance haemodialysis: association with treatment time and dialysis dose. J Nephrol.

[12] Kimata N, Karaboyas A, Bieber BA, Pisoni RL, Morgenstern H, Gillespie BW (2014). Gender, low Kt/V, and mortality in Japanese hemodialysis patients: opportunities for improvement through modifiable practices. Hemodial Int.

[13] Kousoula V, Georgianos PI, Mavromatidis K, Syrganis C, Thodis E, Panagoutsos S (2019). Reversed connection of cuffed, tunneled, dual-lumen catheters with increased blood flow rate maintains the adequacy of delivered dialysis despite the higher access recirculation. Int Urol Nephrol.

[14] Health AAMARCf. Arbor Research Collaborative for Health. DOPPS practice monitor, c2020 [cited 2019 Aug 19]

[15] Ward RA (1999). Blood flow rate: an important determinant of urea clearance and delivered Kt/V. Adv Ren Replace Ther.

[16] Alfurayh O, Galal O, Sobh M, Fawzy M, Taher S, Qunibi W (1993). The effect of extracorporeal high blood flow rate on left ventricular function during hemodialysis–an echocardiographic study. Clin Cardiol.

[17] Trivedi HS, Kukla A, Prowant B, Lim HJ (2007). A study of the extracorporeal rate of blood flow and blood pressure during hemodialysis. Hemodial Int.

[18] Bosch JP, Lew SQ, Barlee V, Mishkin GJ, von Albertini B (2006). Clinical use of high-efficiency hemodialysis treatments: long-term assessment. Hemodial Int.

[19] Cheung AK, Sarnak MJ, Yan G, Berkoben M, Heyka R, Kaufman A (2004). Cardiac diseases in maintenance hemodialysis patients: results of the HEMO Study. Kidney Int.

[20] McClellan WM, Soucie JM, Flanders WD (1998). Mortality in end-stage renal disease is associated with facility-to-facility differences in adequacy of hemodialysis. J Am Soc Nephrol.

[21] Nemeth E, Tuttle MS, Powelson J, Vaughn MB, Donovan A, Ward DM (2004). Hepcidin regulates cellular iron efflux by binding to ferroportin and inducing its internalization. Science.

[22] Yacoub MF, Ferwiz HF, Said F (2020). Effect of Interleukin and Hepcidin in Anemia of Chronic Diseases. Anemia.

[23] Tomosugi N, Kawabata H, Wakatabe R, Higuchi M, Yamaya H, Umehara H (2006). Detection of serum hepcidin in renal failure and inflammation by using ProteinChip System. Blood.

[24] Campostrini N, Castagna A, Zaninotto F, Bedogna V, Tessitore N, Poli A (2010). Evaluation of hepcidin isoforms in hemodialysis patients by a proteomic approach based on SELDI-TOF MS. J Biomed Biotechnol.

[25] van der Putten K, Jie KE, van den Broek D, Kraaijenhagen RJ, Laarakkers C, Swinkels DW (2010). Hepcidin-25 is a marker of the response rather than resistance to exogenous erythropoietin in chronic kidney disease/chronic heart failure patients. Eur J Heart Fail.

[26] van der Weerd NC, Grooteman MP, Bots ML, van den Dorpel MA, den Hoedt CH, Mazairac AH (2012). Hepcidin-25 in chronic hemodialysis patients is related to residual kidney function and not to treatment with erythropoiesis stimulating agents. PLoS One.

[27] Weiss G, Theurl I, Eder S, Koppelstaetter C, Kurz K, Sonnweber T (2009). Serum hepcidin concentration in chronic haemodialysis patients: associations and effects of dialysis, iron and erythropoietin therapy. Eur J Clin Invest.

[28] Galesloot TE, Vermeulen SH, Geurts-Moespot AJ, Klaver SM, Kroot JJ, van Tienoven D (2011). Serum hepcidin: reference ranges and biochemical correlates in the general population. Blood.

[29] Gunnell J, Yeun JY, Depner TA, Kaysen GA (1999). Acute-phase response predicts erythropoietin resistance in hemodialysis and peritoneal dialysis patients. Am J Kidney Dis.

[30] Ifudu O, Feldman J, Friedman EA (1996). The intensity of hemodialysis and the response to erythropoietin in patients with end-stage renal disease. N Engl J Med.

[31] Ko GJ, Obi Y, Tortorici AR, Kalantar-Zadeh K (2017). Dietary protein intake and chronic kidney disease. Curr Opin Clin Nutr Metab Care.

[32] Koenig JS, Fischer M, Bulant E, Tiran B, Elmadfa I, Druml W (1997). Antioxidant status in patients on chronic hemodialysis therapy: impact of parenteral selenium supplementation. Wien Klin Wochenschr.

[33] Fujishima Y, Ohsawa M, Itai K, Kato K, Tanno K, Turin TC (2011). Serum selenium levels are inversely associated with death risk among hemodialysis patients. Nephrol Dial Transplant.

[34] Gugliandolo A, Gangemi C, Calabrò C, Vecchio M, Di Mauro D, Renis M (2016). Assessment of glutathione peroxidase-1 polymorphisms, oxidative stress and DNA damage in sensitivity-related illnesses. Life Sci.

