# Supplementary figures and images for: Effect of increased blood flow rate on renal anemia and hepcidin concentration in hemodialysis patients
Source: BMC Nephrol. 2021 Jun 15;22:221. doi: 10.1186/s12882-021-02426-7 (PMC8204539; doi:10.1186/s12882-021-02426-7)

## Slide 1
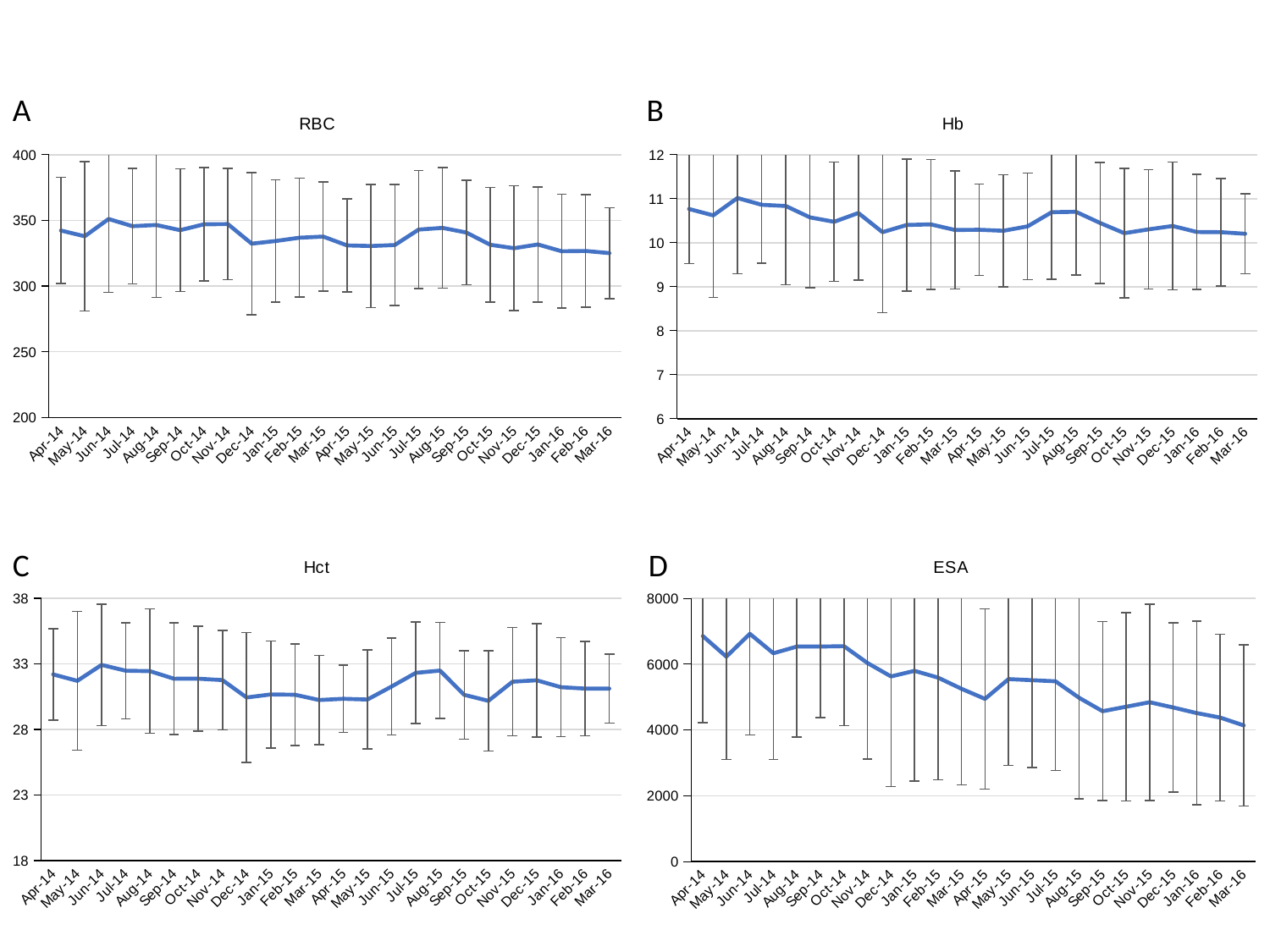

A
B
### Chart: RBC
| Category | |
|---|---|
| 41730 | 342.27272727272725 |
| 41760 | 337.90909090909093 |
| 41791 | 351.09090909090907 |
| 41821 | 345.59090909090907 |
| 41852 | 346.5 |
| 41883 | 342.54545454545456 |
| 41913 | 347.04545454545456 |
| 41944 | 347.1818181818182 |
| 41974 | 332.27272727272725 |
| 42005 | 334.3181818181818 |
| 42036 | 336.8181818181818 |
| 42064 | 337.6818181818182 |
| 42095 | 330.95454545454544 |
| 42125 | 330.5 |
| 42156 | 331.27272727272725 |
| 42186 | 343.0 |
| 42217 | 344.3181818181818 |
| 42248 | 340.77272727272725 |
| 42278 | 331.45454545454544 |
| 42309 | 328.8181818181818 |
| 42339 | 331.6818181818182 |
| 42370 | 326.5 |
| 42401 | 326.72727272727275 |
| 42430 | 325.09090909090907 |
### Chart: Hb
| Category | |
|---|---|
| 41730 | 10.76818181818182 |
| 41760 | 10.622727272727273 |
| 41791 | 11.018181818181818 |
| 41821 | 10.863636363636362 |
| 41852 | 10.836363636363638 |
| 41883 | 10.577272727272726 |
| 41913 | 10.477272727272727 |
| 41944 | 10.677272727272728 |
| 41974 | 10.24090909090909 |
| 42005 | 10.404545454545454 |
| 42036 | 10.418181818181816 |
| 42064 | 10.290909090909091 |
| 42095 | 10.295454545454545 |
| 42125 | 10.272727272727268 |
| 42156 | 10.37272727272727 |
| 42186 | 10.695454545454545 |
| 42217 | 10.704545454545457 |
| 42248 | 10.45 |
| 42278 | 10.21818181818182 |
| 42309 | 10.304545454545455 |
| 42339 | 10.381818181818181 |
| 42370 | 10.245454545454548 |
| 42401 | 10.24090909090909 |
| 42430 | 10.204545454545455 |
### Chart: Hct
| Category | |
|---|---|
| 41730 | 32.195454545454545 |
| 41760 | 31.704545454545453 |
| 41791 | 32.918181818181814 |
| 41821 | 32.477272727272734 |
| 41852 | 32.445454545454545 |
| 41883 | 31.863636363636363 |
| 41913 | 31.86818181818182 |
| 41944 | 31.763636363636362 |
| 41974 | 30.436363636363637 |
| 42005 | 30.66818181818182 |
| 42036 | 30.645454545454548 |
| 42064 | 30.24090909090909 |
| 42095 | 30.336363636363636 |
| 42125 | 30.281818181818185 |
| 42156 | 31.272727272727277 |
| 42186 | 32.31363636363637 |
| 42217 | 32.486363636363635 |
| 42248 | 30.63636363636363 |
| 42278 | 30.181818181818176 |
| 42309 | 31.640909090909087 |
| 42339 | 31.74545454545454 |
| 42370 | 31.222727272727276 |
| 42401 | 31.11363636363637 |
| 42430 | 31.11363636363636 |C
### Chart: ESA
| Category | |
|---|---|
| 41730 | 6852.272727272727 |
| 41760 | 6227.272727272727 |
| 41791 | 6920.454545454545 |
| 41821 | 6329.545454545455 |
| 41852 | 6534.090909090909 |
| 41883 | 6534.090909090909 |
| 41913 | 6545.454545454545 |
| 41944 | 6034.090909090909 |
| 41974 | 5625.0 |
| 42005 | 5795.454545454545 |
| 42036 | 5590.909090909091 |
| 42064 | 5250.0 |
| 42095 | 4943.181818181818 |
| 42125 | 5545.454545454545 |
| 42156 | 5511.363636363636 |
| 42186 | 5477.272727272727 |
| 42217 | 4977.272727272727 |
| 42248 | 4568.181818181818 |
| 42278 | 4704.545454545455 |
| 42309 | 4840.909090909091 |
| 42339 | 4681.818181818182 |
| 42370 | 4511.363636363636 |
| 42401 | 4375.0 |
| 42430 | 4136.363636363636 |D

Supplement: Supplementary file 1 — Additional file 1: Supplementary Figure S1. Monthly changes in RBC (A), Hb (B), and Hct (C) levels and ESA doses (D) [file 12882_2021_2426_MOESM1_ESM.pptx]
